# Diet quality, weight loss, and diabetes incidence in the Diabetes Prevention Program (DPP)

**DOI:** 10.1186/s40795-020-00400-4

**Published:** 2020-12-15

**Authors:** Benjamin T. Allaire, Ashley H. Tjaden, Elizabeth M. Venditti, John W. Apolzan, Dana Dabelea, Linda M. Delahanty, Sharon L. Edelstein, Mary A. Hoskin, Karla A. Temple, Judith Wylie-Rosett, Lindsay M. Jaacks, DPP Research Group

**Affiliations:** 1 RTI International, 3040 Cornwallis Road, Research Triangle Park, NC USA; 2 Department of Epidemiology and Biostatistics, The Biostatistics Center, Milken Institute School of Public Health, George Washington University, Rockville, MD USA; 3 Department of Psychiatry, University of Pittsburgh School of Medicine, Pittsburgh, PA USA; 4 Pennington Biomedical Research Center, Louisiana State University System, Baton Rouge, LA USA; 5 Department of Epidemiology, Colorado School of Public Health, Aurora, CO USA; 6 Harvard Medical School and Massachusetts General Hospital, Boston, MA USA; 7 National Institute of Diabetes and Digestive and Kidney Diseases, Phoenix, AZ USA; 8 Department of Medicine, University of Chicago Medical Center, Chicago, USA; 9 Albert Einstein College of Medicine, Bronx, NY USA; 10 Global Academy of Agriculture and Food Security, The University of Edinburgh, Roslin, UK; 11 Department of Global Health and Population, Harvard T.H. Chan School of Public Health, Boston, USA

**Keywords:** AHEI, Body weight, DPP, Prediabetes, Type 2 diabetes, Lifestyle intervention, Obesity, Diabetes prevention, Dietary pattern, Race

## Abstract

**Background:**

We evaluated whether diet quality is a predictor of weight loss and reduced diabetes risk, independent of caloric intake in the Diabetes Prevention Program (DPP) cohort, a randomized clinical trial of adults at risk for diabetes.

**Methods:**

This secondary analysis included 2914 participants with available data (964 intensive lifestyle (ILS), 977 metformin, 973 placebo). Dietary intake was assessed using a 117-item food frequency questionnaire. Diet quality was quantified using the Alternative Healthy Eating Index 2010 (AHEI). AHEI ranges from 0 to 110, with higher scores corresponding to higher quality diets. ILS participants had greater improvement (*p* < 0.001) in AHEI over 1-year (4.2 ± 9.0) compared to metformin (1.2 ± 8.5) and placebo (1.4 ± 8.4). We examined the association between AHEI change and weight change from baseline to 1-year using linear regression, and that between 1-year AHEI change and incident diabetes, using hazard models over an average 3 years follow-up. Models were evaluated within treatment group and adjusted for relevant characteristics including caloric intake, physical activity, BMI and AHEI. Models testing incident diabetes were further adjusted for baseline fasting and 2 h glucose.

**Results:**

An increase in AHEI score was associated with weight loss in ILS [β per 10-point increase (SE) -1.2 kg (0.3, *p* < 0.001)], metformin [− 0. 90 kg (0.2, *p* < 0.001)] and placebo [− 0.55 kg (0.2, *p* = 0.01)]. However, AHEI change was not associated with incident diabetes in any group before or after adjustment for weight change.

**Conclusions:**

Controlling for weight, diet quality was not associated with diabetes incidence but helps achieve weight loss, an important factor in diabetes prevention.

**Supplementary Information:**

The online version contains supplementary material available at 10.1186/s40795-020-00400-4.

## Background

The role of diet quality in the development of type 2 diabetes is not well understood. The relationship remains challenging to consider due to the paucity of longitudinal data on diet quality and diabetes, and the confounding effect of weight loss. Although diabetes incidence decreases with weight loss, as supported by the Diabetes Prevention Program (DPP) [1], recent work suggests that weight loss alone is not sufficient to fully explain the association between dietary intake and diabetes [2]. There may be an independent effect of diet quality on diabetes risk.

A preponderance of the work on this question has adopted a reductionist approach, investigating the impact of a single food group on diabetes incidence whilst, in most cases, adjusting for total energy intake, physical activity, and body mass index (BMI). Significant reductions in diabetes incidence have been reported with increasing intake of whole grains, green leafy vegetables, and yogurt in meta-analyses of prospective cohort studies [3–7]. Sugar-sweetened beverages and red meat, on the other hand, are associated with a significant increase in diabetes risk in similar meta-analyses of prospective cohort studies [8, 9]. More than single foods or beverages, overall patterns of healthy and unhealthy dietary intake may better predict population health outcomes.

Surprisingly few studies have evaluated the effect of dietary patterns or measures of overall diet quality on diabetes risk [10]. Cespedes et al. (2016) and Qiao et al. (2016) showed higher diet quality scores reduced diabetes incidence in post-menopausal women participating in the Women’s Health Initiative [11, 12]. Recent analyses of the Harvard cohort studies (Nurses’ Health Study [NHS], NHS II, and the Health Professionals Follow-up Study) found that a ≥ 10% increase in the AHEI reduced diabetes incidence by 16% [2].

Most of these studies were in largely homogeneous populations. de Oliveira Otto et al. and Jacobs et al. observed the impact of diet quality differences on diabetes risk for more diverse populations, but only for scores at baseline, not longitudinal changes [13, 14]. Given that dietary patterns have been shown to vary by race/ethnicity [15], it remains to be seen whether or not changes in overall diet quality have an independent effect on diabetes risk in more diverse populations.

The lack of studies relating diet quality to diabetes are due, in part, to measurement issues: there is no single agreed-upon measure of what “optimal” diet quality is. In this paper, we use the Alternative Healthy Eating Index (AHEI) as a proxy for overall diet quality. The AHEI is a composite score of 11 food groups and nutrients including fruits, vegetables, whole grains, sugar-sweetened beverages, nuts and legumes, red/processed meats, sodium, fats (*trans*, polyunsaturated, and long chain fats), and alcohol. We chose to focus on AHEI given that it has the widest score range of any of these scores (and thus is less prone to exposure misclassification), was found to be the most predictive of weight change and chronic disease outcomes including type 2 diabetes in a previous study [16], and to facilitate comparisons to previous studies [11, 12, 17]. The AHEI is highly correlated with other measures of diet quality, such as the Mediterranean diet and the Dietary Approaches to Stop Hypertension (DASH) diet, likely because these scores are generally derived from similar sets of food groups and nutrients.

The objectives of this study were: (1) to estimate the association between changes in AHEI and weight loss, over the first year of intervention and diabetes incidence in the DPP over 3.2 years of follow up, and (2) to determine if the associations differ across race/ethnicity subpopulations. We hypothesized that improved diet quality would be associated with weight loss and decreased diabetes incidence in all race/ethnic subgroups.

## Methods

### Study population

Our sample consists of participants from the DPP. The DPP was a multicenter randomized controlled trial, which enrolled participants at a high risk of developing type 2 diabetes between 1996 and 1999 [18]. A total of 3234 participants were randomized to one of three treatment groups: intensive lifestyle intervention (ILS, *n* = 1079), 850 mg metformin twice daily (*n* = 1073), or placebo (*n* = 1082). The lifestyle intervention used an individually-administered 16-session core curriculum over the first 24 weeks with follow up post-core sessions at least every other month for the remainder of the DPP trial. The intervention followed a standard protocol with the primary intervention goals of achieving and maintaining a weight loss of ≥7% initial body weight and a moderate intensity activity level of ≥150 min per week [19]. Session materials and strategies to reduce fat and calorie intake were tailored to the needs of an ethnically diverse population. Nutrition and corresponding behavior modification session topics addressed dietary fat and calorie self-monitoring, managing cues that shape eating habits, energy balance, and healthy eating out [19]. Four standard calorie (fat-gram) levels were assigned according to baseline weight and designed to produce a 1–2 lb. weight loss per week, with 25% or less of calories from fat. Study protocols were approved by the institutional review boards at all sites and written informed consent was obtained from all participants. This secondary analysis adheres to the STROBE Statement. DPP is registered in ClinicalTrials.gov (NCT00004992).

### Diet quality assessment

Dietary intake was assessed by in-person interview at baseline and year 1 using a 117-item food frequency questionnaire (FFQ) from the Insulin Resistance Atherosclerosis Study (IRAS) FFQ [20], adapted to include ethnic and regional foods expected to represent DPP participants. The IRAS FFQ itself was developed for a culturally diverse US population and was validated against eight 24-h dietary recalls completed over the same 1-year period as the FFQ [21]. For the DPP FFQ, the main body of the questionnaire contained 117 line items (compared to 114 line items on the IRAS FFQ), plus an open-ended query for foods not included within the line items. Foods added to enhance sensitivity to regional and ethnic foods were identified through queries to each of the clinical centers.

The nutrient content of foods was determined using the DietSys Nutrient Analysis Program and Nutrition Data System (version 2.6/8A/23, Nutrition Coordinating Center, University of Minnesota, Minneapolis, MN, USA) by the DPP Nutrition Coding Center at the University of South Carolina. Individual nutrient intake was estimated by using the frequency and portion size of intake for each food item*.* Food groups were assessed using as close to the AHEI assessed groups as was available in the dataset. While the primary analysis for this study includes the data from the baseline and year 1 visit.

We quantified diet quality using AHEI 2010 [22] with one modification to accommodate the DPP dietary data (Supplemental Table 1). Briefly, because DPP did not contain whole grain grams per day, we substituted daily servings of high-fiber grains and breads for daily grams of whole grains. We assumed 5 servings per day for women and 6 for men. These substitutions occurred prior to statistical analyses. AHEI ranges from 0 to 110, with higher scores corresponding to higher quality diets.

### Outcome and covariate assessment

Oral glucose tolerance tests (OGTTs) were completed at annual visits and fasting glucose was obtained at mid-year visits following standardized procedures during DPP. Diabetes incidence was defined as a fasting plasma blood glucose ≥126 mg/dl (7.0 mmol/l) or 2-h post-load plasma glucose ≥200 mg/dl (11.1 mmol/1), confirmed by a repeat test within 6 weeks, according to the 1997 American Diabetes Association criteria [18, 23].

Anthropometric measurements were performed with participants wearing light clothing and without shoes. Body weight was measured in duplicate on a calibrated balance beam scale, zeroed before each measurement. Standing height was determined in duplicate with a calibrated standard stadiometer, with the heels shoulder width apart, in a fully vertical position. Height and weight measurements were used to calculate BMI (kg/m^2^). All staff performing measurements and questionnaires were centrally trained and certified to do so.

Self-reported physical activity was assessed using the Modifiable Activity Questionnaire and expressed as the average metabolic equivalent (MET-hours) per week for the previous year [24] . At the time of DPP randomization, a screening questionnaire was used to obtain age, sex, race/ethnicity, educational attainment, family history of diabetes, history of smoking, and alcohol intake [18].

### Statistical analyses

Changes in AHEI, dietary intake and weight were calculated as year 1 minus the baseline value so that increases are reported as positive numbers. Changes in subscores were compared from baseline to year 1 both visually, by radar plot, and statistically, via paired t-tests. Descriptive statistics are presented as percentages, mean ± SD, or median [Q1, Q3]] for nonnormally distributed data. Comparisons between groups were computed using ANOVA for continuous variables and χ^2^ tests for categorical variables. Except where noted, *P* values< 0.05 were considered nominally statistically significant, with no adjustments made for multiple tests. We examined the association between AHEI score change and weight change from baseline to year 1 using linear regression. We ran three sets of models: (1) a model for all participants; and (2) models stratified by treatment arm (ILS, metformin, and placebo) and (3) stratified by race/ethnicity group. Three sets of linear regression models were run: Model 1 adjusted for potential confounders including baseline values of AHEI, age, sex, BMI, MET hours per week, total daily energy intake as well as change from baseline to year one of MET hours per week and total daily energy intake; Model 2 used Model 1 covariates as well as baseline values of education, smoking status, family history of diabetes, alcohol use, and change from baseline to year one of MET hours and total energy intake; Model 3 also adjusted for baseline values of dietary fiber, carbohydrate, total fat and saturated fat at baseline and change to year 1 of each. Models without treatment arm stratification (i.e. models with all participants or those with race/ethnicity stratification) are adjusted for treatment group, and models without race/ethnicity stratification (i.e. models with all participants or those with treatment arm stratification) were adjusted for race/ethnicity.

We used Cox proportional hazard models to estimate the association of changes in AHEI score with incident diabetes over 3.2 years of follow-up in DPP (hazard ratios [HR] and 95% confidence intervals [CI]). We analyzed all participants combined, adjusted for treatment group, and separately stratified by treatment arm to estimate these associations within the placebo, lifestyle, and metformin groups. We fit models adjusting for baseline values of AHEI, age, sex, race/ethnicity, education, total energy intake, physical activity (MET hours), BMI, fasting and 2 h glucose, family history of diabetes, smoking, and alcohol intake as well change from baseline to year one of physical activity and total energy intake. After assessing the interaction between race/ethnicity and AHEI change, we stratified the analysis by race/ethnicity. We further adjusted for baseline weight and weight change from baseline to year 1 to estimate the direct and indirect effects, via weight change, of AHEI in these models.

Additionally, we followed the approach of Hamman et al. to develop four subgroups based on whether the participant met a weight change target (reduced weight by 5% or more from baseline) and if AHEI increased by 10% from baseline at year 1, plotting the hazard ratios for incident diabetes over 3.2 years of follow-up in DPP for each subgroup [1]. A 5% weight loss threshold was chosen because it is a commonly used threshold in previous studies, including DPP [25]. A 10% increase in AHEI score is equal to 4.4 increase on average for our population. The increase can be nearly achieved, for example, by eliminating sugar-sweetened beverages or by increasing the servings of fruits and vegetables.

To assess whether AHEI score over time predicts long-term weight through DPP (approximately 3.2 years post-randomization), we used mixed linear models for all participants as well as stratified by treatment group and race/ethnicity group. SAS version 9.4 was used for all analyses (SAS Institute, Cary, NC).

## Results

Our analysis included 2914 participants (90.1% of those randomized) with complete baseline and 1-year data (964 ILS, 977 metformin, and 973 placebo). Participants included in the analyses were, on average, middle-aged (mean ± SD, age 50.8 ± 10.6 years) and female (67.5%). Forty-five percent of the sample was minority race/ethnicity; 19% were African American, 16% Hispanic, 5% American Indian, and 4% Asian.

**Fig. 1:**
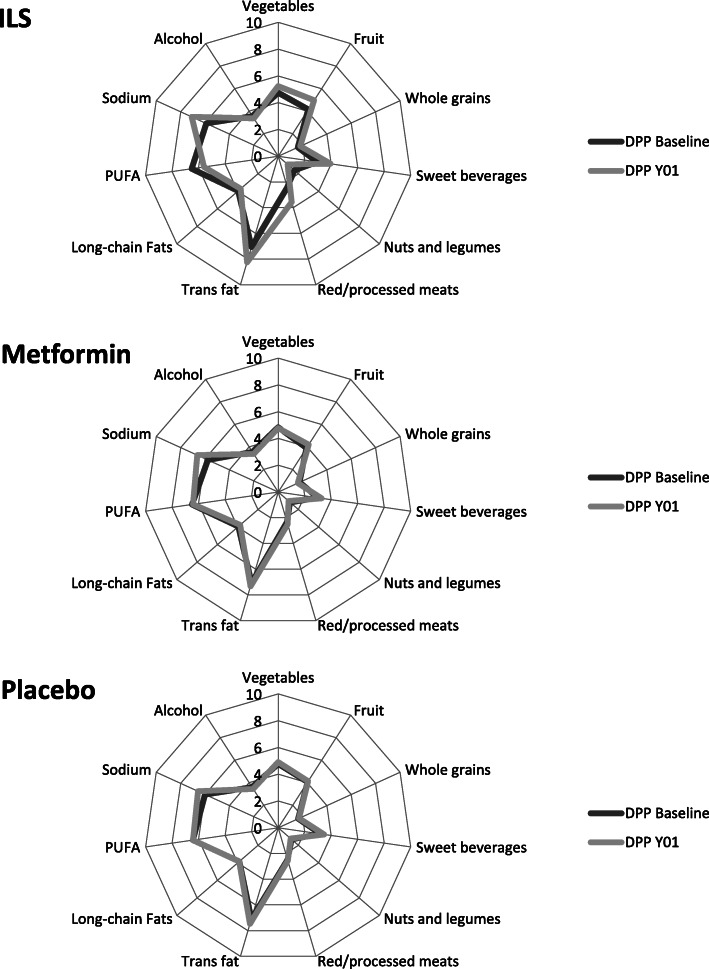
Radar plots of AHEI subscores by treatment group and visit. Note: Each subscore adds 0–10 points to the overall score, resulting in a range of 0–110. Higher scores are associated with greater dietary quality

Mean baseline AHEI and 1-year changes to the AHEI scores by treatment group are presented in Table 1. Baseline AHEI for the entire sample was 44.2 ± 10.4. Although we found no differences in baseline AHEI across treatment groups, ILS participants had greater improvement (*p* < 0.001) in AHEI over 1 year (4.2 ± 9.0 points) compared to metformin (1.2 ± 8.5) and placebo (1.4 ± 8.4) participants. In relative terms, participants saw an increase in AHEI score of approximately 9.5% relative to baseline over 1 the first year of intervention among ILS participants compared to 2.7% among metformin and 3.2% among placebo participants. Increases in AHEI over 1 year were largely driven by participants consuming less sodium, fewer trans fats, and fewer sugar-sweetened beverages (Fig. 1)*.* Paired t-tests (results available upon request) comparing the baseline and year 1 score component values (points contributed) within study group revealed statistically significant changes for all components for the Lifestyle Group (*p* < 0.05). All components except vegetables and alcohol had statistically significant changes within the Metformin group (*p* < 0.05). In the Placebo group, only vegetables, sweet beverages, trans fat and sodium had significant changes from baseline to year 1. Mean baseline AHEI and 1-year changes to the AHEI scores by racial/ethnic group are presented in Supplemental Table 2. Asian participants had the highest mean baseline AHEI (47.6 ± 10.1), more than 10 points higher than the lowest racial/ethnic group (American Indian, 37.2 ± 8.8).

**Table 1:** Baseline Demographics, Participant Characteristics, and AHEI Baseline and Year 1 Scores by treatment arm

	All	ILS	Metformin	Placebo	***p-***value
	N = 2914	***N*** = 964	***N*** = 977	***N*** = 973	
**Demographics**					
Age (years)	50.8 ± 10.6	50.7 ± 11.1	51.3 ± 10.3	50.5 ± 10.3	0.249
Female	1967 (67.5%)	657 (68.2%)	637 (65.2%)	673 (69.2%)	0.151
Race/ethnicity					0.379
% Caucasian	1614 (55.4%)	535 (55.5%)	548 (56.1%)	531 (54.6%)	
% African American	564 (19.4%)	172 (17.8%)	200 (20.5%)	192 (19.7%)	
% Hispanic	460 (15.8%)	157 (16.3%)	151 (15.5%)	152 (15.6%)	
% American Indian	154 (5.3%)	50 (5.2%)	49 (5.0%)	55 (5.7%)	
% Asian	122 (4.2%)	50 (5.2%)	29 (3.0%)	43 (4.4%)	
Education (years)	14.8 ± 3.1	14.8 ± 3.1	14.9 ± 3.1	14.7 ± 3.2	0.579
Current Smoker	181 (6.2%)	50 (5.2%)	61 (6.2%)	70 (7.2%)	0.187
Family History of Diabetes	2018 (69.3%)	665 (69.1%)	674 (69.0%)	679 (69.9%)	0.899
Hypertension^a^	832 (28.6%)	272 (28.2%)	288 (29.5%)	272 (28.0%)	0.728
**Characteristics**					
BMI (kg/m^2^)	33.9 ± 6.6	33.8 ± 6.7	33.8 ± 6.6	34.2 ± 6.7	0.369
Waist (cm)	105.1 ± 14.4	104.9 ± 14.7	104.9 ± 14.4	105.3 ± 14.2	0.760
Waist-to-Hip	0.92 ± 0.09	0.92 ± 0.09	0.93 ± 0.09	0.92 ± 0.08	0.360
Fasting Glucose (mg/dl)	106.4 ± 8.2	106.3 ± 8.1	106.4 ± 8.4	106.6 ± 8.2	0.772
2-Hour Glucose (mg/dl)	164.6 ± 17.0	164.3 ± 16.8	165.0 ± 17.2	164.4 ± 17.1	0.670
HbA1c (%)	5.9 ± 0.5	5.9 ± 0.5	5.9 ± 0.5	5.9 ± 0.5	0.798
Total daily energy intake (kcal)	1901.4 [1459.8, 2565.4]	1911.1 [1445.7, 2513.3]	1916.0 [1485.2, 2607.3]	1878.2 [1445.1, 2559.0]	0.354
Leisure MET hrs per week	9.9 [3.9, 20.7]	9.9 [3.8, 21.3]	10.1 [4.0, 20.8]	9.5 [4.0, 19.4]	0.713
**AHEI Scores**					
DPP Baseline	44.2 ± 10.4	44.4 ± 10.5	43.8 ± 10.3	44.4 ± 10.5	0.327
DPP Year 1 (Y01)	46.4 ± 10.2	48.6 ± 10.0	45.0 ± 10.0	45.9 ± 10.3	<.001
Difference Baseline to Year 1	2.3 ± 8.8	4.2 ± 9.0	1.2 ± 8.5	1.4 ± 8.4	<.001

Abbreviations: *AHEI* Alternative Healthy Eating Index, *BMI* Body Mass Index, *DPP* Diabetes Prevention Program, *HbA1c* Hemoglobin A1c, *ILS* Intensive lifestyle intervention, *MET* Metabolic Equivalent

Data are n (%), mean ± SD, or median [Q1, Q3]

^a^Hypertension is defined as meeting any of three criteria: SBP ≥140 mmHg, diastolic blood pressure (DBP) ≥90 mmHg, or taking medications that lower blood pressure

Change in AHEI from baseline to 1 year was significantly associated with weight loss at year 1 [β per 10-point increase (SE, *p*-value) -0. 89 kg (0.13, *p* < 0.001), Table 2]. Results were consistent across the ILS [− 1.23 kg (0.28, *p* < 0.001)], metformin [− 0.90 kg (0.19, *p* < 0.001)], and placebo [− 0.55 kg (0.20, *p* = 0.01)] groups (p-interaction = 0.083). The interaction term for race/ethnicity and AHEI change was significant (*p* = 0.020), thus the model was stratified by race/ethnicity as well. AHEI change was associated with weight loss, especially in Caucasian [− 1.13 kg (0.19, *p* < 0.001)], Hispanic [− 0.85 kg (0.29, *p* = 0.003)] and American Indian (− 0.90, *p* = 0.156) participants. The effect sizes for African American (− 0.36, *p* = 0.216), and Asian (0.49, *p* = 0.286) participants were much smaller in magnitude and too noisy to detect statistical significance. Additional regression analyses, including a quintile-exposure regression of AHEI on weight change and changing length of follow up to through the end of DPP (an average of 3.2 years of follow-up) to include weight measurements collected at each visit through the end of DPP, revealed the weight change results were linear across the distribution of AHEI scores and robust to weight changes over longer time horizons (Supplemental Tables 3 and 4).

**Table 2:** Effect of change in AHEI (per 10-point increase) on weight change (kg) from baseline to year 1 among participants of the Diabetes Prevention Program (n = 2914)

	N	Beta Coefficient	Standard Error	***p-***value
Model 1: All participants^a,b^	2914	−0.991	0.133	<.0001
Model 2: All participants^a,b^	2914	−0.890	0.133	<.0001
Model 3: All participants ^a,b^	2914	−0.512	0.155	0.001
**Stratified Models (Model 2)**				
*Treatment group (p-interaction = 0.083)*				
Lifestyle ^b^	964	−1.232	0.278	<.0001
Metformin^b^	977	−0.896	0.194	<.0001
Placebo^b^	973	−0.547	0.201	0.007
*Racial/ethnic group (p-interaction = 0.020)*				
Caucasian^a^	1614	−1.128	0.191	<.0001
African American^a^	564	−0.363	0.293	0.216
Hispanic^a^	460	−0.852	0.287	0.003
American Indian^a^	154	−0.901	0.631	0.155
Asian^a^	122	+ 0.494	0.461	0.286

Model 1: Linear regression models adjusted for baseline values of AHEI, age, sex, BMI, MET hours per week, total energy intake as well as change from baseline to year one of MET hours per week and total energy intake

Model 2: Linear regression models adjusted for Model 1 covariates as well as baseline values of education, smoking status, family history of diabetes, alcohol use

Model 3: Also adjusted for baseline values of dietary fiber, carbohydrate, total fat and saturated fat at baseline and change to year 1 of each

^a^Also adjusted for treatment group

^b^Also adjusted for race/ethnicity

Change in AHEI from baseline to 1 year did not statistically significantly predict incident diabetes over approximately 3 years of follow-up, in models without weight change (HR [95% CI] per 10-point increase, 0.92 [0.82–1.03]) or with weight change (0.98 [0.87–1.10]) (Table 3). Separate models by treatment groups were not qualitatively different; HRs (95% CIs) were 1.01 (0.79–1.30) in ILS, 0.96 (0.78–1.19) in metformin, and 0.98 (0.83–1.16) in placebo with adjustment for weight change over the same year period (p-interaction = 0.959).

**Table 3:** Effect of AHEI change (per 10-point increase) from baseline to year 1 on time to diabetes over 3.2 years of follow-up in DPP using Cox proportional hazard models

	N	Model 1 Adjusted Hazard Ratios (95% CI)	Model 2 Adjusted Hazard Ratios (95% CI)	Model 3 Adjusted Hazard Ratios (95% CI)
All participants ^a,b^	2914	0.903 (0.811, 1.006)	0.919 (0.822, 1.026)	0.980 (0.874, 1.098)
**Stratified Models**				
*Treatment group*		*p-interaction =* 0.9785	*p-interaction = 0.9591*	*p-interaction = 0.9726*
Lifestyle ^b^	964	0.884 (0.704, 1.109)	0.856 (0.677, 1.082)	1.013 (0.789, 1.301)
Metformin^b^	977	0.869 (0.715, 1.058)	0.915 (0.745, 1.123)	0.963 (0.783, 1.185)
Placebo^b^	973	0.933 (0.796, 1.093)	0.957 (0.810, 1.130)	0.981 (0.829, 1.162)
*Racial/ethnic group*		*p-interaction =* 0.1269	*p-interaction = 0.0636*	*p-interaction = 0.1391*
Caucasian^a^	1614	**0.840 (0.727, 0.970)**	**0.834 (0.716, 0.972)**	0.899 (0.767, 1.055)
African American^a^	564	1.246 (0.967, 1.607)	1.238 (0.948, 1.615)	1.275 (0.981, 1.657)
Hispanic^a^	460	0.881 (0.682, 1.138)	0.951 (0.727, 1.244)	1.043 (0.788, 1.382)
American Indian^a^	154	1.161 (0.636, 2.119)	0.603 (0.302, 1.207)	0.662 (0.333, 1.317)
Asian^a^	122	0.622 (0.336, 1.148)	1.376 (0.656, 2.886)	1.422 (0.666, 3.034)

Model 1: Cox proportional hazard models adjusted for baseline values of AHEI, age, sex, BMI, MET hours, and total energy intake

Model 2: Also adjusted for baseline values of education, smoking status, family history of diabetes, alcohol use, fasting and 2 h glucose as well as change from baseline to year one of MET hours per week and total energy intake

Model 3: Also adjusted for baseline weight and weight change from baseline to year 1

^a^Also adjusted for treatment group

^b^Also adjusted for race/ethnicity

We plotted the adjusted HRs in Supplemental Fig. 1 to visually assess the impact of improvements in AHEI and weight on time to diabetes, dichotomized by AHEI change > 10% versus ≤ 10% and weight change of > 5% versus ≤5% to create four distinct groups. Overall, 40.1% of participants achieved a ≥ 10% increase in AHEI. Among those who achieved < 5% weight loss, only 34.5% achieved a ≥ 10% increase in AHEI whereas among those who achieved ≥5% weight loss, 50.5% achieved a ≥ 10% increase in AHEI. Weight change was the driver of diabetes onset: the largest effects occur when participants lost more than 5% weight from baseline where the adjusted HR (95% CI) falls from 0.98 (0.80–1.19) to 0.52 (0.39–0.70). Supplemental Fig. 1 presents results for all treatment groups. We also ran analyses within the ILS treatment group, who lost the most weight, and found that the relationship held within this treatment group as well (Supplemental Fig. 2).

## Discussion

We report small improvements in overall diet quality over the first year of a randomized controlled trial for the prevention of type 2 diabetes, particularly among those in the ILS arm. These changes in AHEI were associated with statistically significant weight loss across all treatment arms with the greatest improvements in the ILS arm (− 1.2 kg per 10-point change in AHEI compared to − 0.5 kg in the placebo arm). However, we did not find a significant association between changes in AHEI and incident type 2 diabetes. These results are consistent with those found in Sylvetsky et al. (2017) who found that high-fiber carbohydrates, and lower total and saturated fat intake predicted weight loss when adjusted for changes in calorie intake, yet these categories did not predict reductions in diabetes incidence [26]. Our estimates of whole grains, an AHEI subscore category, and fats, which are represented by three AHEI subscores, are closely associated with, but do not comprise all of the change in AHEI score that led to weight loss in this study. Further adjustment for baseline and change in dietary fiber, carbohydrate, total fat and saturated fat intake did not negate the relationship between change in AHEI score and weight loss, as evidenced in Table 2.

Though the overall suggestion of eating less fat and fewer calories often results in improvements in food choices and a more balanced eating pattern, the ILS intervention sessions addressed diet quality/food choices/healthy eating habits directly in only one session, independent of weight loss goals, and modest changes in eating patterns were thus observed [19, 27]. The ongoing intervention for this group involved individual strategies further emphasized healthy eating behaviors to achieve weight loss goals. Diet changes were observed in the lifestyle intervention arm [27, 28], which included significantly higher fruit intake and lower red meat, dairy, and sweets intake. These changes were consistent not only with a lower-fat diet, but also an overall healthier diet reflective of the Food Guide Pyramid. Our results suggest that diet quality helps achieve weight loss, even after adjustment for caloric intake and physical activity, and that practitioners should also emphasize components of an overall healthful diet to maximize weight loss. This is consistent with the 2015–2020 Dietary Guidelines for Americans, which emphasize overall dietary patterns rather than specific macro- or micronutrients [29].

We observed an increase in AHEI score of approximately 9.5% relative to baseline over 1 year of intervention among ILS participants compared to 2.7% among metformin and 3.2% among placebo participants. Content analysis showed that these improvements were largely driven by participants consuming less sodium and trans fat, and fewer sugar-sweetened beverages. This may have been due to the sessions on dietary fat, calorie balance, and healthy eating out. The results among the placebo group (average increase of 1.4 points over 1 year) are larger than increases observed in the U.S. general population using the 1999–2000 NHANES data. The AHEI for Americans increased by 0.69 points per year on average with reductions in consumption of trans fats intake accounting for more than half of that improvement [30].

AHEI had impacts on the weight of Caucasian (− 1.1 kg per 10-point increase in AHEI) and Hispanic (− 0.9 kg per 10-point increase in AHEI) participants. We did not find a statistically significant association for any other racial/ethnic groups. This is consistent with the fact that Caucasian and Hispanic participants had the greatest increase in AHEI from baseline to 1 year: mean increase of 2.3 and 3.0, respectively, compared to mean increases ranging from 1.5 to 1.9 among American Indian, African American, and Asian participants. These findings agree with previous work on the dietary patterns of the DPP by race, which found significant reductions in dietary fat for Hispanics [27]. A previous analysis of three prospective cohorts (NHS, NHS II, and the Health Professionals Follow-up Study) found that each SD increase in AHEI score was associated with a − 0.47 kg decrease in weight [31], which is very similar to the − 0.5 kg decrease in weight for each SD increase in AHEI score among DPP placebo participants in our study. In a more diverse prospective cohort of Americans (Multi-ethnic Study of Atherosclerosis), adherence to better dietary quality was associated with a statistically significant 0.54-unit decrease in follow-up BMI among Caucasian participants and a 0.43-unit decrease in follow-up BMI among Chinese participants, with similar reductions among Hispanic participants (0.57-unit decrease, *p* = 0.15), though they were not statistically significant, and there was no association among African American participants (0.08-unit decrease, *p* = 0.44) [32]. These findings are largely consistent with our findings of the strongest effects of AHEI on weight loss among Caucasians. However, these results were found using the Health Eating Index (HEI), which is measure of adherence to dietary guidelines not chronic disease risk. The correlation between the indices is between 0.54–0.65 [17].

We did not find a statistically significant effect of diet quality as assessed by AHEI on diabetes incidence, either independent or mediated through weight loss. Even among participants who had greater than a 10% increase in AHEI from baseline to year 1, if sufficient weight loss was not also achieved (e.g., at least 5%), there was no significant reduction in diabetes incidence. This finding is not consistent with one previous study that analyzed three prospective cohorts (NHS, NHS II, and the Health Professionals Follow-up Study) and found that a greater than 10% increase in AHEI reduced diabetes incidence by 16% [2]. Our data are substantially different than NHS, given that they are the result of randomized controlled trial and represent more diverse race/ethnic groups than the NHS. They also observed that just 32% of the association between AHEI and diabetes could be attributed to changes in body weight [2]. One explanation may be that the effects of AHEI on diabetes incidence may only hold for Caucasians. Our results, though still not significant, were much stronger for Caucasians. The findings could imply that AHEI may need further validation as a measure of diet quality in non-Caucasians.

In addition, we observed that the increases in AHEI were largely driven by participants consuming less sodium, fewer trans fats, and fewer sugar-sweetened beverages. While meta-analyses support a strong, consistent association between sugar-sweetened beverage intake and obesity and type 2 diabetes [8], sodium and trans fat are more strongly associated with hypertension, heart disease and stroke [33, 34]. The consistent signal for an association between AHEI and weight loss in our study is promising, but more research is needed to understand impacts on subsequent type 2 diabetes, particularly the relationship between race/ethnicity, diet quality, and diabetes incidence.

Several scores have been proposed to quantify diet quality including the AHEI, the HEI, the DASH diet score, and Mediterranean-style diet scores. Both DASH diet scores and Mediterranean-style diets have been associated with reduced risk of type 2 diabetes in prospective studies [35–38]. However, the AHEI and ILS curriculum were developed at different times: AHEI was first developed in 2010 whereas the ILS curriculum was developed in 1995. The ILS curriculum focused on weight loss through a lower-fat diet to reduce overall energy intake [19]. The ILS curriculum also included a discussion of the importance of eating more grains, vegetables, and fruit, but did not include behavioral goals related to the intake of these food groups. The AHEI scores allot up to 30 points for eating healthier types of fat, but the DPP curriculum emphasized total fat reductions and did not emphasize the delineate between healthier fats initially including intake of sources of omega-3 fatty acids or PUFA which would have been consistent with maximizing AHEI scores. Data limitations required us to substitute servings of high fiber breads and grains for servings of whole grains, However, our whole grain servings were consistent with the national levels of whole grain servings found in Wang et al. (2014), which mitigates this concern [30].

Strengths of our study included the prospective design, longitudinal assessment of dietary intake, use of a validated FFQ and estimation of total caloric intake, the diverse sample population with high retention rates, and the gold standard definition of incident type 2 diabetes rather than relying on self-report as has been done in previous studies [2]. To the best of our knowledge, this is the first of the seminal diabetes prevention program studies to evaluate changes in using a comprehensive diet quality measure such as AHEI.

Our study, however, is not without limitations. First, our study is based upon the DDP population. To the extent that the DDP population does not reflect the demographic characteristics of the United States, this constrains the generalizability of our findings. Second, due to incomplete FFQ data, we substituted daily servings of high-fiber grains and breads for daily grams of whole grains. Our AHEI scores for these categories were in line with population averages calculated by Wang et al. (2014). (27) Finally, we note that the DDP lifestyle coaching did not specifically address eating diets associated with a higher AHEI score. Although, counseling was offered promoting the consumption of fewer calories and a lower fat diet.

## Conclusions

Many lifestyle interventions for the prevention of type 2 diabetes, including the DPP, emphasize very specific nutritional advice such as reductions in dietary fat intake to achieve weight loss, which may explain why maintenance of these behaviors over time has proven difficult [39, 40]. Our results suggest that overall diet quality captured by 11 components (higher consumption of vegetables, fruits, high-fiber grains and bread, nuts and legumes, long-chain omega-3 fatty acids, and PUFAs, and lower consumption of red/processed meat, sugar-sweetened beverages and fruit juice, trans fat, and sodium) from the AHEI score is associated with significantly greater weight loss among participants in a randomized controlled trial in the first year. This adds to the growing literature supporting strong associations between overall diet quality and weight loss, the latter generally one of the strongest predictors of type 2 diabetes [23, 31, 32]. Practitioners should provide practical advice centered on these dietary components to promote healthful diets and weight loss.

## Supplementary Information

**Additional file 1: **CONSORT Diagram outlining participant data availability for this analysis. **Supplemental Table 1.** Summary of AHEI calculation and modifications from Chiuve et al. AHEI-2010. **Supplemental Table 2** Baseline Demographics, Participant Characteristics, and AHEI Baseline and Year 1 Scores by Race/Ethnicity**. Supplemental Figure 1.** Hazard ratios (HRs) for diabetes onset over 3.2 years of follow-up in DPP defined by meeting thresholds of percentage change at 1 year compared with those meeting neither of the goals (group 1). **Supplemental Figure 2.** Hazard ratios (HRs) for diabetes onset over 3.2 years of follow-up in DPP defined by meeting thresholds of percentage change at 1 year compared with those meeting neither of the goals (group 1), among ILS participants only. **Supplemental Table 3.** Associations of AHEI change quintile on weight change from baseline to year 1 among participants of the Diabetes Prevention Program (*n* = 2914). **Supplemental Table 4.** Effect of change in AHEI (per 10-point increase) from baseline to year 1 on weight change (kg) over 3.2 years of follow-up in DPP (n = 2914).

## Data Availability

In accordance with the NIH Public Access Policy, we continue to provide all manuscripts to PubMed Central including this manuscript DPP/DPPOS has provided the protocols and lifestyle and medication intervention manuals to the public through its public website (https://www.dppos.org). The DPPOS abides by the NIDDK data sharing policy and implementation guidance as required by the NIH/NIDDK (https://www.niddkrepository.org/studies/dppos/). All data are available through the NIDDK Data Repository (https://repository.niddk.nih.gov/studies/dppos/).

## Abbreviations

AHEI: Alternative Healthy Eating Index
DPP: Diabetes Prevention Program
DPPOS: Diabetes Prevention program Outcomes Study
ILS: Intensive Lifestyle
