# Supplementary material for: Diet quality, weight loss, and diabetes incidence in the Diabetes Prevention Program (DPP)
Source: BMC Nutr. 2020 Dec 15;6:74. doi: 10.1186/s40795-020-00400-4 (PMC7737274; doi:10.1186/s40795-020-00400-4)
Supplement: Supplementary file 1 — Additional file 1: CONSORT Diagram outlining participant data availability for this analysis. Supplemental Table 1. Summary of AHEI calculation and modifications from Chiuve et al. AHEI-2010. Supplemental Table 2 Baseline Demographics, Participant Characteristics, and AHEI Baseline and Year 1 Scores by Race/Ethnicity. Supplemental Figure 1. Hazard ratios (HRs) for diabetes onset over 3.2 years of follow-up in DPP defined by meeting thresholds of percentage change at 1 year compared with those meeting neither of the goals (group 1). Supplemental Figure 2. Hazard ratios (HRs) for diabetes onset over 3.2 years of follow-up in DPP defined by meeting thresholds of percentage change at 1 year compared with those meeting neither of the goals (group 1), among ILS participants only. Supplemental Table 3. Associations of AHEI change quintile on weight change from baseline to year 1 among participants of the Diabetes Prevention Program (n = 2914). Supplemental Table 4. Effect of change in AHEI (per 10-point increase) from baseline to year 1 on weight change (kg) over 3.2 years of follow-up in DPP (n = 2914). [file 40795_2020_400_MOESM1_ESM.docx]

**Online Supplemental Material**

**prepared for**

**Diet Quality, Weight Loss, and Diabetes Incidence in the**

**Diabetes Prevention Program (DPP)**

Benjamin T. Allaire, MS, Ashley H. Tjaden, MPH, Elizabeth M. Venditti PhD, John W. Apolzan, PhD, Dana Dabelea, MD, PhD, Linda M. Delahanty, Sharon L. Edelstein, ScM, Mary A. Hoskin, MS, RD, Karla A. Temple, PhD, RDN Judith Wylie-Rosett, EdD, Lindsay M. Jaacks, PhD, DPP Research Group

**CONSORT Diagram outlining participant data availability for this analysis**

**3234**

Participants randomized

Lifestyle

**1079**

Metformin

**1073**

Placebo

**1082**

Baseline FFQ Completed: **1054**

Baseline FFQ Completed: **1057**

Baseline FFQ Completed:

**1064**

Year 1 FFQ Completed: **964**

Year 1 FFQ Completed: **977**

Year 1 FFQ Completed: **973**

**Supplemental Table 1. Summary of AHEI calculation and modifications from Chiuve et al AHEI-2010**

| **Component** | **Criteria for minimum score (0)** | **Criteria for maximum score (10)** |
| --- | --- | --- |
| Vegetables, *servings/day* | 0 | ≥5 |
| Fruit, *servings/day* | 0 | ≥4 |
| Whole grains, *servings/day* ^1^ | 0 |  |
| Women |  | 4 |
| Men |  | 5 |
| Sugar-sweetened beverages and fruit juice, *servings/day* | ≥1 | 0 |
| Nuts and legumes, *servings/day* | 0 | ≥1 |
| Red/processed meat, *servings/day* | ≥1.5 | 0 |
| *trans* Fat,*% of energy* | ≥4 | ≤0.5 |
| Long-chain (n-3) fats (EPA + DHA), *mg/d* | 0 | 250 |
| PUFA,*% of energy* | ≤2 | ≥10 |
| Sodium, *mg/day* | Highest decile | Lowest decile |
| Alcohol, d*rinks/d* |  |  |
| Women | ≥2.5 | 0.5–1.5 |
| Men | ≥3.5 | 0.5–2.0 |
| Total | 0 | 110 |
| ^1^ Original AHEI score used daily servings of whole grains, which was not available in DPP/DPPOS. Instead, daily servings of high-fiber grains and breads was used. | | |

| **Supplemental Table 2** Baseline Demographics, Participant Characteristics, and AHEI Baseline and Year 1 Scores by Race/Ethnicity**.** | | | | | | | |
| --- | --- | --- | --- | --- | --- | --- | --- |
|  | **All** | **Caucasian** | **African Am** | **Hispanic** | **Am Indian** | **Asian** |  |
|  | **N=2914** | **N=1614** | **N=564** | **N=460** | **N=154** | **N=122** | **p-value** |
| **Demographics** |  |  |  |  |  |  |  |
| Age (years) | 50.8 ± 10.6 | 52.3 ± 10.8 | 50.4 ± 10.0 | 48.6 ± 10.0 | 44.3 ± 9.7 | 51.0 ± 9.8 | <.001 |
| Female (n, %) | 1967 (67.5%) | 1051 (65.1%) | 421 (74.6%) | 308 (67.0%) | 136 (88.3%) | 51 (41.8%) | <.001 |
| Education (years) | 14.8 ± 3.1 | 15.5 ± 2.6 | 14.8 ± 2.9 | 12.6 ± 4.0 | 13.3 ± 2.6 | 15.8 ± 2.8 | <.001 |
| Current Smoker (n, %) | 181 (6.2%) | 70 (4.3%) | 60 (10.6%) | 35 (7.6%) | 11 (7.1%) | 5 (4.1%) | <.001 |
| Family Hx Diab (n, %) | 2018 (69.3%) | 1089 (67.6%) | 412 (73.0%) | 318 (69.1%) | 115 (74.7%) | 84 (68.9%) | 0.087 |
| Hypertension* (n, %) | 832 (28.6%) | 460 (28.5%) | 202 (35.8%) | 101 (22.0%) | 21 (13.6%) | 48 (39.3%) | <.001 |
| **Characteristics** |  |  |  |  |  |  |  |
| BMI (kg/m^2^) | 33.9 ± 6.6 | 34.1 ± 6.8 | 35.3 ± 7.0 | 33.2 ± 5.7 | 33.2 ± 5.8 | 29.1 ± 4.4 | <.001 |
| Waist (cm) | 105.1 ± 14.4 | 106.3 ± 14.8 | 106.7 ± 14.5 | 101.4 ± 12.7 | 104.7 ± 12.5 | 95.3 ± 10.8 | <.001 |
| Waist-to-Hip | 0.92 ± 0.09 | 0.93 ± 0.09 | 0.92 ± 0.09 | 0.92 ± 0.08 | 0.92 ± 0.08 | 0.94 ± 0.08 | 0.359 |
| Fasting Glucose (mg/dl) | 106.4 ± 8.2 | 106.7 ± 8.2 | 107.7 ± 8.1 | 106.0 ± 8.0 | 99.5 ± 7.9 | 107.0 ± 7.5 | <.001 |
| 2-Hour Glucose (mg/dl) | 164.6 ± 17.0 | 164.9 ± 16.8 | 164.5 ± 17.8 | 163.4 ± 16.8 | 163.4 ± 17.0 | 166.3 ± 17.0 | 0.306 |
| HbA1c (%) | 5.9 ± 0.5 | 5.8 ± 0.4 | 6.2 ± 0.6 | 5.9 ± 0.5 | 5.9 ± 0.4 | 6.0 ± 0.4 | <.001 |
| Total energy intake (kcal) | 1901.4  [1459.8 , 2565.4] | 1892.4  [1471.3 , 2486.0] | 1782.0  [1381.3 , 2476.5] | 2097.6  [1582.8 , 2942.7] | 2204.2  [1558.4 , 3033.8] | 1676.9  [1267.7 , 2300.4] | <.001 |
| Leisure MET Hrs | 9.9  [3.9 , 20.7] | 10.2  [4.1 , 20.2] | 9.5  [3.2 , 18.9] | 8.1  [3.5 , 20.3] | 15.7  [4.8 , 29.4] | 10.2  [3.7 , 20.7] | <.001 |
| **AHEI Scores** |  |  |  |  |  |  |  |
| DPP Baseline | 44.2 ± 10.4 | 44.8 ± 10.5 | 42.7 ± 9.9 | 45.3 ± 10.3 | 37.2 ± 8.8 | 47.6 ± 10.1 | <.001 |
| DPP Year 1 | 46.4 ± 10.2 | 47.1 ± 10.3 | 44.6 ± 9.5 | 48.3 ± 10.1 | 38.7 ± 7.5 | 49.5 ± 9.2 | <.001 |
| Difference Baseline to Year 1 | 2.3 ± 8.8 | 2.3 ± 8.7 | 1.8 ± 8.3 | 3.0 ± 9.7 | 1.5 ± 8.5 | 1.9 ± 8.2 | 0.214 |

Data are n (%), mean±SD, or median [Q1, Q3]. *Hypertension is defined as meeting any of three criteria: SBP ≥140 mmHg, diastolic blood pressure (DBP) ≥90 mmHg, or taking medications that lower blood pressure

**Supplemental Figure 1.** Hazard ratios (HRs) for diabetes onset over 3.2 years of follow-up in DPP defined by meeting thresholds of percentage change at 1 year compared with those meeting neither of the goals (group 1).


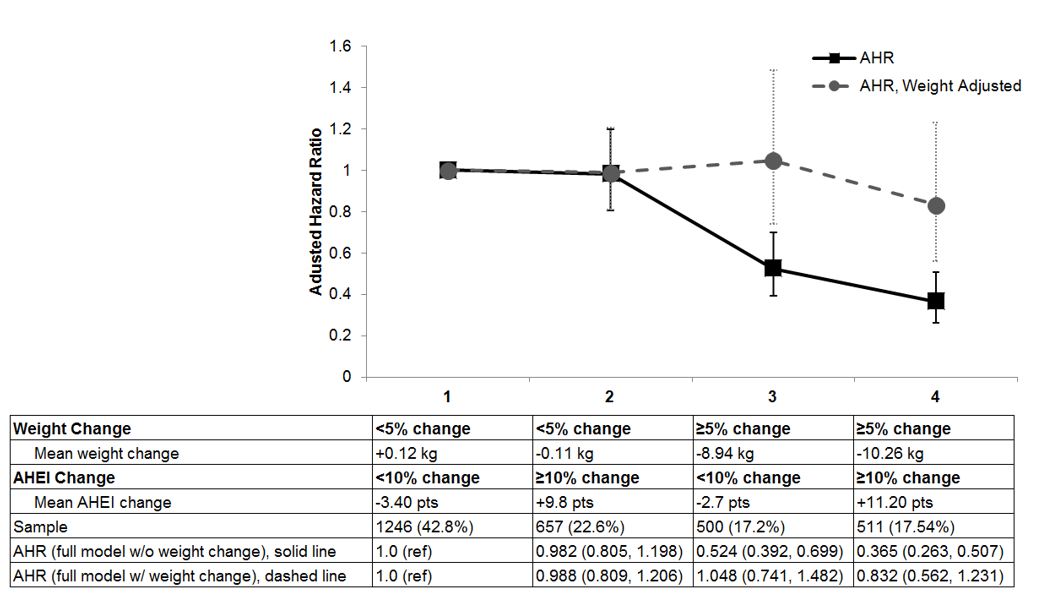


All treatment groups combined. The solid line is adjusted for baseline covariates (age, sex, BMI, MET hours, total energy intake, fasting and 2hr glucose) as well as change from baseline to year 1 of MET hours and total energy intake; the dashed line is also adjusted for weight change as a continuous measure from baseline to year 1 within group. Without weight change, the effect of dietary quality is statistically significant. When it is added to the model, the statistical significance goes away. Solid line, adjusted HRs (AHRs) (full model without weight change); Dashed line, AHRs for full model with weight change included.

**Supplemental Figure 2.**

Hazard ratios (HRs) for diabetes onset over 3.2 years of follow-up in DPP defined by meeting thresholds of percentage change at 1 year compared with those meeting neither of the goals (group 1), among ILS participants only.


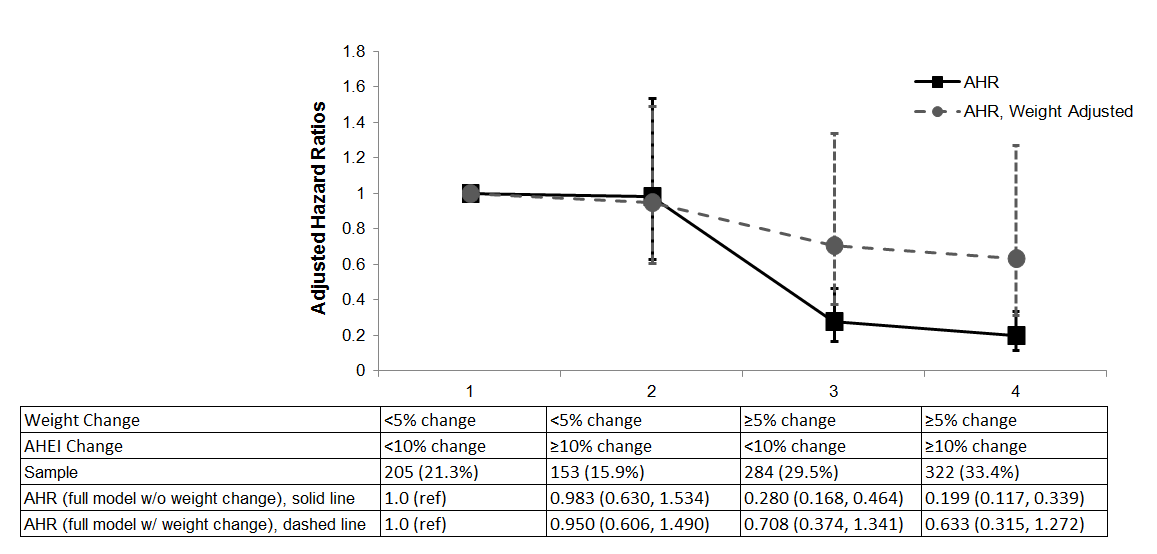


Among ILS participants. The solid line is adjusted for baseline covariates (age, sex, BMI, MET hours, total energy intake, fasting and 2-hr glucose) as well as change from baseline to year 1 of MET hours and total energy intake; the dashed line is also adjusted for weight change as a continuous measure from baseline to year 1 within group. Without weight change, the effect of dietary quality is statistically significant. When it is added to the model, the statistical significance goes away. Solid line, adjusted HRs (AHRs) (full model without weight change); Dashed line, AHRs for full model with weight change included.

**Supplemental Table 3.**

Associations of AHEI change quintile on weight change from baseline to year 1 among participants of the Diabetes Prevention Program (n=2914).

| **Quintile** | **N** | **Mean AHEI Change** | **Beta Coefficient** | **Standard Error** | **p-value** |
| --- | --- | --- | --- | --- | --- |
| 1st | 582 | -9.79 points | 0.000 (ref) | Ref | Ref |
| 2nd | 583 | -2.06 points | -0.401 | 0.331 | 0.2264 |
| 3rd | 583 | +2.14 points | -0.404 | 0.342 | 0.2378 |
| 4th | 583 | +6.43 points | -1.328 | 0.347 | 0.0001 |
| 5th | 583 | +14.54 points | -2.357 | 0.359 | <0.0001 |

Linear regression models adjusted for treatment group, baseline values of AHEI, age, sex, race/ethnicity, education, smoking status, family history of diabetes, alcohol use, BMI, MET hours, total energy intake as well as change from baseline to year one of AHEI, MET hours, and total energy intake.

**Supplemental Table 4.**

Effect of change in AHEI (per 10-point increase) from baseline to year 1 on weight change (kg) over 3.2 years of follow-up in DPP (n=2914).

|  | **Beta Coefficient** | **Standard Error** | **p-value** |
| --- | --- | --- | --- |
| All participants*† | -0.597 | 0.070 | <0.0001 |
| **Stratified Models** |  |  |  |
| *Treatment group* |  |  |  |
| Lifestyle† | -0.740 | 0.141 | <0.0001 |
| Metformin† | -0.335 | 0.095 | 0.0005 |
| Placebo† | -0.255 | 0.080 | 0.0016 |
| *Racial/ethnic group* |  |  |  |
| Caucasian* | -0.688 | 0.110 | <0.0001 |
| African American* | -0.324 | 0.127 | 0.0108 |
| Hispanic* | -0.856 | 0.139 | <.0001 |
| American Indian* | -0.103 | 0.242 | 0.6716 |
| Asian* | -0.158 | 0.1652 | 0.3408 |

Mixed linear regression models adjusted for baseline values of AHEI, age, sex, education, smoking status, family history of diabetes, alcohol use, BMI, MET hours, total energy intake. *Also adjusted for treatment group. †Also adjusted for race/ethnicity.

**Pennington Biomedical Research Center**

**(Baton Rouge, LA)**

George A. Bray, MD*

Kishore Gadde, MD*

Iris W. Culbert, BSN, RN, CCRC**

Jennifer Arceneaux RN, BSN**

Annie Chatellier, RN, CCRC**

Amber Dragg RD, LDN**

Catherine M. Champagne, PhD, RD

Crystal Duncan, LPN

Barbara Eberhardt, RD, LDN

Frank Greenway, MD

Fonda G. Guillory, LPN

April A. Herbert, RD

Michael L. Jeffirs, LPN

Betty M. Kennedy, MPA

Erma Levy, RD

Monica Lockett, LPN

Jennifer C. Lovejoy, PhD

Laura H. Morris, BS

Lee E. Melancon, BA, BS

Donna H. Ryan, MD

Deborah A. Sanford, LPN

Kenneth G. Smith, BS, MT

Lisa L. Smith, BS

Julia A. St.Amant, RTR

Richard T. Tulley, PhD

Paula C. Vicknair, MS, RD

Donald Williamson, PhD

Jeffery J. Zachwieja, PhD

**University of Chicago (Chicago, IL)**

Kenneth S. Polonsky, MD*

Janet Tobian, MD, PhD*

David A. Ehrmann, MD*

Margaret J. Matulik, RN, BSN**

Bart Clark, MD

Kirsten Czech, MS

Catherine DeSandre, BA

Ruthanne Hilbrich, RD

Wylie McNabb, EdD

Ann R. Semenske, MS, RD

**Jefferson Medical College (Philadelphia, PA)**

Jose F. Caro, MD*

Kevin Furlong, DO*

Barry J. Goldstein, MD, PhD*

Pamela G. Watson, RN, ScD*

Kellie A. Smith, RN, MSN**

Jewel Mendoza, RN, BSN**

Wendi Wildman, RN**

Renee Liberoni, MPH

John Spandorfer, MD

Constance Pepe, MS, RD

**University of Miami (Miami, FL)**

Richard P. Donahue, PhD*

Ronald B. Goldberg, MD*

Ronald Prineas, MD, PhD*

Jeanette Calles, MSEd**

Juliet Ojito, RN**

Patricia Rowe, MPA**

Paul Cassanova-Romero, MD

Sumaya Castillo-Florez, MPH

Hermes J. Florez, MD

Anna Giannella, RD, MS**

Lascelles Kirby, MS

Carmen Larreal

Olga Lara

Valerie McLymont, RN

Jadell Mendez

Arlette Perry, PhD

Patrice Saab, PhD

Beth Veciana

**The University of Texas Health Science Center**

**(San Antonio, TX)**

Steven M. Haffner, MD, MPH*

Helen P. Hazuda, PhD*

Maria G. Montez, RN, MSHP, CDE**

Kathy Hattaway, RD, MS

Carlos Lorenzo, MD, PhD

Arlene Martinez, RN, BSN, CDE

Tatiana Walker, RD, MS, CDE

**University of Colorado (Denver, CO)**

Dana Dabelea, MD, PhD*

Richard F. Hamman, MD, DrPH*

Patricia V. Nash, MS**

Sheila C. Steinke, MS**

Lisa Testaverde, MS**

Denise R. Anderson, RN, BSN

Larry B. Ballonoff, MD

Alexis Bouffard, MA, RN, BSN

Brian Bucca OD, FAOO

B. Ned Calonge, MD, MPH

Lynne Delve

Martha Farago, RN

James O. Hill, PhD

Shelley R. Hoyer, BS

Tonya Jenkins, RD, CDE

Bonnie T. Jortberg, MS, RD, CDE

Dione Lenz, RN, BSN, CDE

Marsha Miller, MS, RD

Leigh Perreault, MD

David W. Price, MD

Judith G. Regensteiner, PhD

Helen Seagle, MS, RD

Carissa M. Smith, BS

Brent VanDorsten, PhD

**Joslin Diabetes Center (Boston, MA)**

Edward S. Horton, MD*

Kathleen E. Lawton, RN**

Catherine S. Poirier, RN, BSN**

Kati Swift, RN, BSN**

Ronald A. Arky, MD

Marybeth Bryant

Jacqueline P. Burke, BSN

Enrique Caballero, MD

Karen M. Callaphan, BA

Barbara Fargnoli, RD

Therese Franklin

Om P. Ganda, MD

Ashley Guidi, BS

Mathew Guido, BA

Sharon D. Jackson, MS, RD, CDE

Alan M. Jacobsen, MD

Lori Lambert, MS, RD, LD

Sarah Ledbury, Med, RD

Margaret Kocal, RN, CDE

Lyn M. Kula, RD

Maureen A. Malloy, BS

Maryanne Nicosia, MS, RD

Cathryn F. Oldmixon, RN

Jocelyn Pan, BS, MPH

Marizel Quitingon

Stacy Rubtchinsky, BS

Jessica Sansoucy, BS

Dana Schweizer, BSN

Ellen W. Seely, MD

Donald Simonson, MD

Fannie Smith, MD

Caren G. Solomon, MD, MPH

Jeanne Spellman, RD

James Warram, MD

**VA Puget Sound Health Care System and University of Washington (Seattle, WA)**

Steven E. Kahn, MB, ChB*

Brenda K. Montgomery, RN, BSN, CDE**

Wilfred Fujimoto, MD

Robert H. Knopp, MD

Edward W. Lipkin, MD

Michelle Marr, BA

Ivy Morgan-Taggart

Anne Murillo, BS

Dace Trence, MD

Lonnese Taylor, RN, BS

April Thomas, RD, MPH, CDE

Elaine C. Tsai, MD, MPH

**University of Tennessee (Memphis, TN)**

Samuel Dagogo-Jack, MD, MSc, FRCP, FACP*

Abbas E. Kitabchi, PhD, MD, FACP*

Mary E. Murphy, RN, MS, CDE, MBA**

Laura Taylor, RN, BSN, CDE**

Jennifer Dolgoff, RN, BSN**

William B. Applegate, MD, MPH

Michael Bryer-Ash, MD

Debra Clark, LPN

Sandra L. Frieson, RN

Uzoma Ibebuogu, MD

Raed Imseis, MD

Helen Lambeth, RN, BSN

Lynne C. Lichtermann, RN, BSN

Hooman Oktaei, MD

Harriet Ricks

Lily M.K. Rutledge, RN, BSN

Amy R. Sherman, RD, LD

Clara M. Smith, RD, MHP, LDN

Judith E. Soberman, MD

Beverly Williams-Cleaves, MD

**Northwestern University’s Feinberg School of Medicine (Chicago, IL)**

Boyd E. Metzger, MD*

Mark E. Molitch, MD*

Mariana K. Johnson, MS, RN**

Daphne T. Adelman, MBA, RN

Catherine Behrends

Michelle Cook, MS

Marian Fitzgibbon, PhD

Mimi M. Giles, MS, RD

Deloris Heard, MA

Cheryl K.H. Johnson, MS, RN

Diane Larsen, BS

Anne Lowe, BS

Megan Lyman, BS

David McPherson, MD

Samsam C. Penn, BA

Thomas Pitts, MD

Renee Reinhart, RN, MS

Susan Roston, RN, RD

Pamela A. Schinleber, RN, MS

**Massachusetts General Hospital (Boston, MA)**

David M. Nathan, MD*

Charles McKitrick, BSN**

Heather Turgeon, BSN**

Mary Larkin, MSN**

Kathy Abbott

Ellen Anderson, MS, RD

Laurie Bissett, MS, RD

Kristy Bondi, BS

Enrico Cagliero, MD

Jose C. Florez, MD, PhD+

Kali D’Anna

Linda Delahanty, MS, RD

Valerie Goldman, MS, RD

Peter Lou, MD

Alexandra Poulos

Elyse Raymond, BS

Christine Stevens, RN

Beverly Tseng

**University of California-San Diego (La Jolla, CA)**

Jerrold M. Olefsky, MD*

Elizabeth Barrett-Connor, MD*

Mary Lou Carrion-Petersen, RN, BSN**

Madeline Beltran, RN, BSN, CDE

Lauren N. Claravall, BS

Jonalle M. Dowden, BS

Steven V. Edelman, MD

Robert R. Henry, MD

Javiva Horne, RD

Marycie Lamkin, RN

Simona Szerdi Janesch, BA

Diana Leos

Sunder Mudaliar, MD

William Polonsky, PhD

Jean Smith, RN

Jennifer Torio-Hurley

Karen Vejvoda, RN, BSN, CDE, CCRC

**Columbia University (New York, NY)**

F. Xavier Pi-Sunyer, MD*

Jane E. Lee, MS**

David B. Allison, PhD

Nnenna Agharanya

Nancy J. Aronoff, MS, RD

Maria Baldo

Jill P. Crandall, MD

Sandra T. Foo, MD

Susan Hagamen, MS, RN, CDE

Jose A. Luchsinger, MD, MPH

Carmen Pal, MD

Kathy Parkes, RN

Mary Beth Pena, RN

Ellen S. Rooney, BA

Gretchen E.H. Van Wye, MA

Kristine A. Viscovich, ANP

**Indiana University (Indianapolis, IN)**

David G. Marrero, PhD*

Kieren J. Mather, MD*

Melvin J. Prince, MD*

Susie M. Kelly, RN, CDE**

Marcia A. Jackson**

Gina McAtee**

Paula Putenney, RN**

Ronald T. Ackermann, MD

Carolyn M. Cantrell

Yolanda F. Dotson, BS

Edwin S. Fineberg, MD

Megan Fultz

John C. Guare, PhD

Angela Hadden

James M. Ignaut, MA

Marion S. Kirkman, MD

Erin O’Kelly Phillips

Beverly D. Porter, MSN

Paris J. Roach, MD

Nancy D. Rowland, BS, MS

Madelyn L. Wheeler, RD

**Medstar Research Institute (Washington, DC)**

Vanita Aroda, MD*

Robert E. Ratner, MD*

Gretchen Youssef, RD, CDE**

Sue Shapiro, RN, BSN, CCRC**

Catherine Bavido-Arrage, MS, RD, LD

Geraldine Boggs, MSN, RN

Marjorie Bronsord, MS, RD, CDE

Ernestine Brown

Wayman W. Cheatham, MD

Susan Cola

Cindy Evans

Peggy Gibbs

Tracy Kellum, MS, RD, CDE

Renee Wiggins, RD

Milvia Lagarda

Lilia Leon

Claresa Levatan, MD

Milajurine Lindsay

Asha K. Nair, BS

Maureen Passaro, MD

Angela Silverman

Gabriel Uwaifo, MD

Debra Wells-Thayer, NP, CDE

**University of Southern California/UCLA Research Center (Alhambra, CA)**

Mohammed F. Saad, MD*

Karol Watson, MD*

Maria Budget**

Sujata Jinagouda, MD**

Medhat Botrous, MD**

Khan Akbar, MD

Claudia Conzues

Perpetua Magpuri

Kathy Ngo

Amer Rassam, MD

Debra Waters

Kathy Xapthalamous

**Washington University (St. Louis, MO)**

Julio V. Santiago, MD*

Samuel Dagogo-Jack, MD, MSc, FRCP, FACP*

Neil H. White, MD, CDE*

Angela L. Brown, MD*

Samia Das, MS, MBA, RD, LD**

Prajakta Khare-Ranade, MSc, RDN, LD**

Tamara Stich, RN, MSN, CDE**

Ana Santiago, RN

Edwin Fisher, PhD

Emma Hurt, RN

Tracy Jones, RN

Michelle Kerr, RD

Lucy Ryder, RN

Cormarie Wernimont, RD, LD

**Johns Hopkins School of Medicine**

**(Baltimore, MD)**

Sherita Hill Golden, MD, MHS, FAHA*

Christopher D. Saudek, MD*

Vanessa Bradley, BA**

Emily Sullivan, MEd, RN**

Tracy Whittington, BS**

Caroline Abbas

Adrienne Allen

Frederick L. Brancati, MD, MHS

Sharon Cappelli

Jeanne M. Clark, MD

Jeanne B. Charleston, RN, MSN

Janice Freel

Katherine Horak, RD

Alicia Greene

Dawn Jiggetts

Deloris Johnson

Hope Joseph

Kimberly Loman

Henry Mosley

John Reusing

Richard R. Rubin, PhD

Alafia Samuels, MD

Thomas Shields

Shawne Stephens

Kerry J. Stewart, EdD

LeeLana Thomas

Evonne Utsey

Paula Williamson

**University of New Mexico (Albuquerque, NM)**

David S. Schade, MD*

Karwyn S. Adams, RN, MSN**

Janene L. Canady, RN, CDE**

Carolyn Johannes, RN, CDE**

Claire Hemphill, RN, BSN**

Penny Hyde, RN, BSN**

Leslie F. Atler, PhD

Patrick J. Boyle, MD

Mark R. Burge, MD

Lisa Chai, RN

Kathleen Colleran, MD

Ysela Gonzales

Doris A. Hernandez-McGinnis

Patricia Katz, LPN

Carolyn King, Med

Amer Rassam, MD

Sofya Rubinchik, MD

Willette Senter, RD

Debra Waters, PhD

**Albert Einstein College of Medicine (Bronx, NY)**

Jill Crandall, MD*

Harry Shamoon, MD*

Janet O. Brown, RN, MPH, MSN**

Gilda Trandafirescu, MD**

Elsie Adorno, BS

Liane Cox, MS, RD

Helena Duffy, MS, C-ANP

Samuel Engel, MD

Allison Friedler, BS

Angela Goldstein, FNP-C, NPP, CSW

Crystal J. Howard-Century, MA

Jennifer Lukin, BA

Stacey Kloiber, RN

Nadege Longchamp, LPN

Helen Martinez, RN, MSN, FNP-C

Dorothy Pompi, BA

Jonathan Scheindlin, MD

Elissa Violino, RD, MS

Elizabeth A. Walker PhD, RN

Judith Wylie-Rosett, EdD, RD

Elise Zimmerman, RD, MS

Joel Zonszein, MD

**University of Pittsburgh (Pittsburgh, PA)**

Trevor Orchard, MD*

Rena R. Wing, PhD*

Susan Jeffries, RN, MSN**

Gaye Koenning, MS, RD**

M. Kaye Kramer, BSN, MPH**

Marie Smith, RN, BSN**

Susan Barr, BS

Catherine Benchoff

Miriam Boraz, PhD

Lisa Clifford, BS

Rebecca Culyba, BS

Marlene Frazier

Ryan Gilligan, BS

Stephanie Guimond, BS

Susan Harrier, MLT

Louann Harris, RN

Andrea Kriska, PhD

Qurashia Manjoo, MD

Monica Mullen, MHP, RD

Alicia Noel, BS

Amy Otto, PhD

Jessica Pettigrew, CMA

Debra Rubinstein, MD

Linda Semler, MS, RD

Cheryl F. Smith, PhD

Elizabeth Venditti, PhD

Valarie Weinzierl, MPH

Katherine V. Williams, MD, MPH

Tara Wilson, BA

**University of Hawaii (Honolulu, HI)**

Richard F. Arakaki, MD*

Renee W. Latimer, BSN, MPH** Narleen K. Baker-Ladao, BS**

Mae K. Isonaga, RD, MPH**

Ralph Beddow, MD

Nina E. Bermudez, MS

Lorna Dias, AA

Jillian Inouye, RN, PhD

Marjorie K. Mau, MD

John S. Melish, MD

Kathy Mikami, BS, RD

Pharis Mohideen, MD

Sharon K. Odom, RD, MPH

Raynette U. Perry, AA

Robin E. Yamamoto, CDE, RD

**Southwest American Indian Centers**

**(Phoenix, AZ; Shiprock, NM; Zuni, NM)**

William C. Knowler, MD, DrPH*+

Norman Cooeyate**

Mary A. Hoskin, RD, MS**

Carol A. Percy, RN, MS**

Alvera Enote**

Camille Natewa**

Kelly J. Acton, MD, MPH

Vickie L. Andre, RN, FNP

Rosalyn Barber

Shandiin Begay, MPH

Peter H. Bennett, MB, FRCP

Mary Beth Benson, RN, BSN

Evelyn C. Bird, RD, MPH

Brenda A. Broussard, RD, MPH, MBA, CDE

Brian C. Bucca, OD, FAAO

Marcella Chavez, RN, AS

Sherron Cook

Jeff Curtis, MD

Tara Dacawyma

Matthew S. Doughty, MD

Roberta Duncan, RD

Charlotte Dodge

Cyndy Edgerton, RD

Jacqueline M. Ghahate

Justin Glass, MD

Martia Glass, MD

Dorothy Gohdes, MD

Wendy Grant, MD

Robert L. Hanson, MD, MPH

Ellie Horse

Louise E. Ingraham, MS, RD, LN

Merry Jackson

Priscilla Jay

Roylen S. Kaskalla

David Kessler, MD

Kathleen M. Kobus, RNC-ANP

Jonathan Krakoff, MD

Jason Kurland, MD

Catherine Manus, LPN

Cherie McCabe

Sara Michaels, MD

Tina Morgan

Yolanda Nashboo

Julie A. Nelson, RD

Steven Poirier, MD

Evette Polczynski, MD

Christopher Piromalli, DO

Mike Reidy, MD

Jeanine Roumain, MD, MPH

Debra Rowse, MD

Robert J. Roy

Sandra Sangster, RD

Janet Sewenemewa

Miranda Smart

Darryl Tonemah, PhD

Rachel Williams, FNP

Charlton Wilson, MD

Michelle Yazzie

**George Washington University Biostatistics Center (DPP Coordinating Center Rockville, MD)**

Raymond Bain, PhD*

Sarah Fowler, PhD*

Marinella Temprosa, PhD*

Michael D. Larsen, PhD*

Tina Brenneman**

Sharon L. Edelstein, ScM**

Solome Abebe, MS

Julie Bamdad, MS

Melanie Barkalow

Joel Bethepu, MPH

Tsedenia Bezabeh, MS

Nicole Butler, MPH

Jackie Callaghan

Caitlin E. Carter, MPH

Costas Christophi, PhD

Gregory M. Dwyer, MPH

Mary Foulkes, PhD

Yuping Gao

Robert Gooding

Adrienne Gottlieb

Kristina L. Grimes

Nisha Grover-Fairchild, MPH

Lori Haffner, MS

Heather Hoffman, PhD

Kathleen Jablonski, PhD

Steve Jones

Tara L. Jones

Richard Katz, MD

Preethy Kolinjivadi, MS

John M. Lachin, ScD

Yong Ma, PhD

Pamela Mucik

Robert Orlosky

Qing Pan, PhD

Susan Reamer

James Rochon, PhD

Alla Sapozhnikova

Hanna Sherif, MS

Charlotte Stimpson

Ashley Hogan Tjaden, MPH

Fredricka Walker-Murray

**Lifestyle Resource Core**

Elizabeth M. Venditti, PhD*

Andrea M. Kriska, PhD

Linda Semler, MS, RD, LDN

Valerie Weinzierl, MPH

**Central Biochemistry Laboratory (Seattle, WA)**

Santica Marcovina, PhD, ScD*

Jessica Harting**

F. Alan Aldrich**

John Albers, PhD

Greg Strylewicz, PhD

**NIH/NIDDK (Bethesda, MD)**

R. Eastman, MD

Judith Fradkin, MD

Sanford Garfield, PhD

Christine Lee, MD, MS

**Centers for Disease Control & Prevention**

**(Atlanta, GA)**

Edward Gregg, PhD

Ping Zhang, PhD

**Nutrition Coding Center (Columbia, SC)**

Elizabeth Mayer-Davis, PhD*

Robert R. Moran, PhD**
